# “French Phage Network” Annual Conference—Seventh Meeting Report

**DOI:** 10.3390/v15020495

**Published:** 2023-02-10

**Authors:** Olivier Schiettekatte, Elsa Beurrier, Luisa De Sordi, Anne Chevallereau

**Affiliations:** 1 Palais de la Découverte, Universcience, 75008 Paris, France; 2 MIVEGEC, Université Montpellier, CNRS, IRD, 34090 Montpellier, France; 3 Centre de Recherche St Antoine, Sorbonne Université, INSERM, 75012 Paris, France; 4 Institut Cochin, Université Paris Cité, CNRS, INSERM, 75014 Paris, France

**Keywords:** bacteriophages, mobile genetic elements, bacterial immunity, ecology and evolution, phage therapy and biotechnology, phage–host interaction, France

## Abstract

The French Phage Network (Phages.fr) has continuously grown since its foundation, eight years ago. The annual conference, held at the Institut Pasteur in Paris, attracted 164 participants from the 11th to the 13th of October 2022. Researchers from academic laboratories, hospitals and private companies shared their ongoing projects and breakthroughs in the very institute where Felix d’Hérelle developed phage therapy over a century ago. The conference was divided into four thematic sessions, each opened by a keynote lecture: “Interaction between phages, mobile genetic elements and bacterial immune system,” “Ecology and evolution of phage–bacteria interactions,” “Molecular interplay between phages and their hosts” and “Therapeutic and biotechnological applications of phages.” A total of 32 talks and 33 posters were presented during the conference.

## 1. Introduction

Investigations on viruses of bacteria, or bacteriophages (phages), started in the early 20th century with their use against pathogenic bacteria [1]. Soon after, the biology of phage–host interactions attracted scientists from different disciplines and provided both the keys to understand major biological processes, from the identification of DNA as the heritable genetic material [2] to gene regulation [3], as well as the tools for the advent of molecular and synthetic biology, such as restriction enzymes [4] or CRISPR-Cas systems [5,6]. More recently, the increasing discovery of novel prokaryotic defence mechanisms has given more credit to the existence of a prokaryotic immune system, which shares striking similarities with the eukaryotic immune system [7]. Prokaryotic defence systems can be directed against, and carried by, their predators and parasites, represented by mobile genetic elements (MGEs) [8,9,10,11].

**Figure 1:**
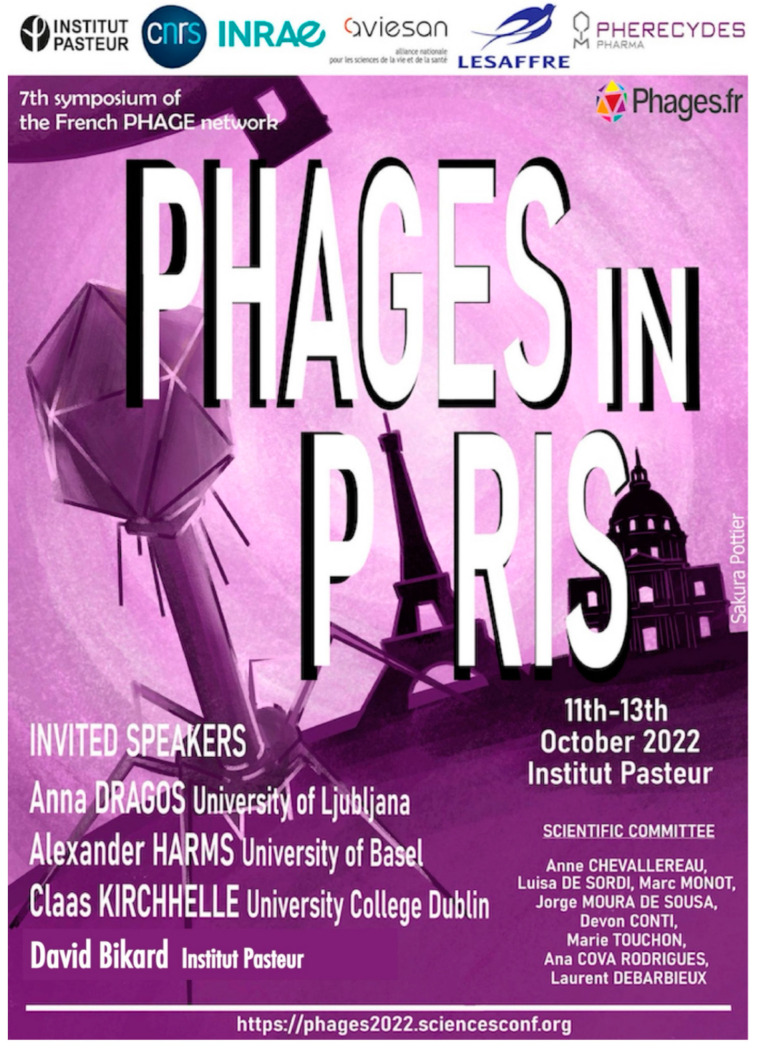
Poster of the conference “Phages in Paris.”

The annual conferences of the French Phage Network aim at gathering researchers, mostly located in France, who study viruses of microbes, their biology, and their applications. Since its creation in 2015, the network has been continuously expanding, reflecting the growing interest in phage research that is observed worldwide. This meeting gives the opportunity to welcome new research groups to the network and to discuss and initiate new collaborations. In 2022, the French Phage Network met at the Institut Pasteur in Paris, in the historical “Emile Duclaux” amphitheatre, from the 11th to the 13th of October 2022 (Figure 1). The meeting gathered 164 participants, of whom one third were students and postdoctoral researchers. The participants were mainly from France (126/164), but 12 other countries were also represented (Belgium, Denmark, Germany, Ireland, Israel, Poland, Senegal, Slovenia, Spain, Switzerland, the UK and the USA). Gender parity was attained among both the speakers (17 men, 16 women) and the attendees (51% women, 49% men). About 11% of the participants were affiliated with private companies. Reflecting the current phage research landscape in France, this year’s meeting was divided into four sessions: “Interaction between phages, mobile genetic elements and bacterial immune system,” “Ecology and evolution of phage–bacteria interactions,” “Molecular interplay between phages and their hosts” and “Therapeutic and biotechnological applications of phages.” Each session was opened by a keynote lecture by invited speakers: Anna Dragoš (University of Ljubljana, Slovenia), Alexander Harms (ETH Zürich, Switzerland), Claas Kirchhelle (University College Dublin, Ireland) and David Bikard (Institut Pasteur Paris, France). Two “flash-talk” sessions allowed the 33 poster presenters to advertise their work, which was discussed during coffee breaks and two poster sessions (Table S1).

## 2. Summary of the Scientific Sessions

### 2.1. Interaction between Phages, Mobile Genetic Elements and Bacterial Immune System

The conference opened with a session dedicated to the interactions among phages, their parasites and the immune system of their bacterial host.

Eugen Pfeifer (postdoctoral researcher, Institut Pasteur, France) shed light on a poorly known class of MGEs, coined phage–plasmids. Phage–plasmids, such as coliphages P1 and N15, are elements that replicate as plasmids, that is, as extrachromosomal circular DNA molecules, but their horizontal transmission is mediated by virions. By developing a computational screen to detect phage–plasmids, Pfeifer and colleagues established that these elements are prevalent (almost 4% of phages and 6% of plasmids in databases) and are found in more than 80 different host genera among Gram-positive and -negative bacteria [12]. Phage–plasmids can have an important impact on the evolution of their hosts, as they often carry antibiotic resistance genes (ARGs), although less frequently than bona fide plasmids. These ARGs can be spread from one bacterial host to another by phage–plasmids upon lysogenic conversion [13]. Another class of MGEs that has a remarkable impact on bacterial evolution is that of Phage Inducible Chromosomal Islands (PICIs). PICIs are genetic elements integrated in bacterial genomes that must hijack capsids produced by helper phages for their dissemination and are, therefore, classified as phage satellites [14]. Because of this parasitism, PICIs often interfere with the life cycle of their helper phages, which can have beneficial consequences on the survival and evolution of their bacterial host. Rodrigo Ibarra-Chavez (postdoctoral researcher, University of Copenhagen, Denmark) reported that PICI-mediated interference promotes the survival of recipient bacteria and reduces the production of helper phage particles or lysogens. This PICI-mediated protection against phage lysis enhances the frequency of transductants within the host population, which favours genetic diversification of bacteria [15]. However, not all phage satellites interfere with the replication of their helper phages. Frédérique Le Roux (researcher, Ifremer, Station Biologique de Roscoff, France) and her team revealed the discovery of a novel family of satellites in *Vibrio*, coined PICMI (Phage Inducible Chromosomal Minimal Islands), which contain a maximum of 12 genes. The mobilization of PICMIs is relatively frequent, and they can be transduced in strains that are resistant to a virulent helper phage, as long as the helper phages can adsorb. Interestingly, PICMIs can interfere with a narrow range of non-helper, virulent phages.

Besides interfering with phage replication, phage satellites can also protect their host by encoding defence systems. David Bikard (researcher, Institut Pasteur, France) and his team discovered hotspots of anti-phage defence systems encoded in *Escherichia coli* phages P2 and their P4 satellites, which enabled the characterisation of a novel abortive infection defence system, named PARIS (Phage Anti Restriction-Induced System). PARIS is activated by the anti-defence proteins that some phages deploy to circumvent restriction/modification or BREX defence systems. Strikingly, the defence systems carried by P4 satellites do not block the infection of their P2 helper phages but instead restrict a broad range of virulent phages. Through this mechanism, P4 satellites can benefit their helper phage, changing our view of satellites as simple phage parasites [16].

Altogether, the speakers have highlighted the important impact of MGEs in bacterial evolution, as they can favour horizontal gene transfer (HGT), notably by serving themselves as vehicles of genes, and also because they can protect their host against phage predation. While the relationships between satellites and their phages are often parasitic, recent findings suggest they are more along a parasitic–mutualistic continuum, as satellites can enhance the reproducibility of their helper phages and, hence, their own dissemination.

These tripartite phage–satellite–host interactions are nonetheless possibly costly for the bacterial host and have prompted the evolution of numerous bacterial defence systems. In past years, myriad novel defence systems were discovered, hinting that many more remain to be found. Giuseppina Mariano (researcher, Newcastle University, UK) reported the identification of a novel defence system in *Pseudomonas aeruginosa*, named Shield, of which the core component, ShdA, is a nuclease harbouring a RmuC domain and is critical for anti-phage activity [17]. François Rousset (postdoctoral researcher, Weizmann Institute of Science, Israel) presented the latest advances on the characterisation of CBASS defence systems, which produce cyclic nucleotide messengers upon sensing phage infection to activate effectors that can disrupt the bacterial membrane, degrade nucleic acids or deplete NAD molecules, leading to cell death. He described novel CBASS effectors that degrade ATP molecules and discovered additional defence systems that also rely on ATP nucleosidases, called Detocs (defence two-component systems) [18].

In response to this diversity of bacterial defence systems, phages have evolved counter-defence measures to maintain their infectivity. One example is that of anti-CRISPR (Acr) proteins that inhibit the adaptive immune system CRISPR-Cas. Acrs are small, very diverse proteins for which computational identification is challenging because they generally share no or little homology to one another. Despite these difficulties, guilt-by-association approaches, with experimentally identified Acrs or with the more conserved Aca (anti-CRISPR–associated) proteins, allowed the identification of new Acrs in the past. Yuvaraj Bhoobalan (postdoctoral researcher, University of Copenhagen, Denmark) proposed a novel method to identify Acr proteins based on the fact that *acr* genes are highly expressed very early during viral infection and, therefore, may be associated with optimal, conserved promoters. The application of this method to the genomes of archaeal viruses allowed the discovery of a novel type I-A Acr inhibitor and putative new Aca proteins, which could enable further discoveries of novel Acrs. This high production of Acrs upon virus entry, which is critical for Acr activity, may also constitute an Achilles’ heel in the virus infection strategy. Indeed, Benoit Pons (postdoctoral researcher, University of Exeter, United Kingdom) tested the hypothesis that translation inhibitors, such as antibiotics, could reduce the infectivity of phages encoding Acr proteins. He consistently found that the amplification of phages encoding Acr proteins was compromised in the presence of sub-inhibitory concentrations of antibiotics, which therefore protected the bacterial host population from lysis [19].

### 2.2. Ecology and Evolution of Phage–Bacteria Interactions

Our knowledge of viral biodiversity has rapidly progressed over the past decade, with the multiplication of metagenomic studies. However, this vast territory still requires ample exploration. Focusing on anaerobic fermenters, Ariane Bize (researcher, Université Paris-Saclay, INRAE, France) reported the search for viruses of methanogenic archaea. She discriminated viruses from active and nonactive hosts by coupling stable isotope probing methods with shotgun metagenomics and identified a set of new viral genomes representing a new viral family, *Speroviridae,* as well as quasi-complete genomes of spindle-shaped viruses targeting methanogenic archaea.

Exploring the role of viruses in shaping microbial communities is challenging, especially in structured and heterogeneous environments such as soil. Sungeun Lee (postdoctoral researcher, Laboratoire Ampère, Ecole Centrale de Lyon, France) aims to identify active virus–host interactions in soil by following the transfer of carbon from autotrophic hosts to their viruses. This method revealed which microbial and viral communities were active, depending on the ecological conditions to which the soils were exposed (e.g., under methane incubation or nitrifying conditions), and allowed the identification of specific virus–host interactions [20,21]. Quentin Lamy-Besnier (postdoctoral researcher, Institut Pasteur, Université Paris Cité) presented a different approach to obtain information on phage–bacteria interactions in complex microbial communities. He applied chromosome conformation capture (3C), a method detecting physical interactions within or between DNA molecules, to study the dynamics of a microbial community composed of 12 bacterial species within the mouse gut. This approach, combined with virome sequencing, allowed identification of 13 functional prophages (amongst 44 predicted), of which 11 were induced in the gut. These results highlight the fact that the gut environment can strongly affect prophage induction, compared with in vitro conditions [22].

The dynamics of (pro)phages in natural environments are key to understanding the evolution of bacteria. Charlène Sagrillo (Ph.D. student, CNRS UMR7564, Université de Lorraine, France) focused on transduction, an important mechanism of HGT mediated by phages, within microbial communities in river water. She studied the distribution of antibiotic resistance genes in bacterial and phage fractions present upstream and downstream of a wastewater treatment plant. Quantification of genes encoding 16S rRNAs, 10 ARGs and 9 MGEs in phage fractions revealed that transducing phages carried up to 16.3% of 16S rRNAs encoding genes in the effluent of the plant (compared with less than 0.1% in rivers). While wastewater was found to be enriched in ARGs, no significant difference in ARGs could be detected in the water downstream of the discharge point of the treatment plant compared with samples collected upstream [23]. In addition to fuelling HGT, phages can influence the evolution of bacteria through the selective pressure they impose on their hosts. Olaya Rendueles (researcher, Institut Pasteur, Paris, France) studied the long-term costs and benefits associated with prophage presence in *Klebsiella pneumoniae.* Coevolution of a lysogenic strain with a non-lysogenic phage-sensitive strain led to the rapid emergence of phage resistance in the non-lysogenic population through the loss of the capsule. Interestingly, new resistant clones emerged at later time points; they remained capsulated but with reduced capacity to adsorb phages [24]. While phage–bacteria coexistence can result from reciprocal coevolution, Yoann Anciaux (postdoctoral researcher, Institut des Sciences de l’Évolution de Montpellier, France) showed that phage–bacteria coexistence can be achieved in the absence of evolution. He studied the epidemiological dynamics between *E. coli* and phage T7 by fluoro-luminometric measurements over 4–5 days and found that the bacterial population goes through cycles of growth–collapse–regrowth without the emergence of phage-resistant bacteria. These results underline the need to refine mechanistic and theoretical models (e.g., classical compartmental epidemiological models) to better explain phage–bacteria epidemiological dynamics.

Harnessing the power of phages to drive the evolution of their hosts, Alfonso Jaramillo (researcher, i2SysBio, CSIC-University of Valencia, Spain) proposed biotechnological applications of phages for the directed evolution of proteins and RNAs. Using phages M13, T7 and P2 and their *E. coli* host enabled the engineering of different transcription factors and a riboswitch [25]. This phage-based methodology allows for accelerated directed evolution of proteins and RNAs.

### 2.3. Molecular Interplay between Phages and Their Host

One of the most remarkable ways for phages to modify their host biology is through lysogenic conversion, where prophage genes provide their bacterial hosts with new physiological functions, which can be beneficial. However, prophage acquisition can also come with fitness costs, notably when it comes to survival in stressful conditions. Pauline Misson (Ph.D., INRAE MICALIS, Jouy-en-Josas, France) investigated the roles of prophages in the capacity of their bacterial host to survive in macrophages. She followed the induction of an active prophage, called Gally, in adherent-invasive *E. coli* strain LF82. She unexpectedly found that Gally transcription was repressed when LF82 multiplied inside macrophages, thus suggesting that LF82 “tames” its prophages to survive in this stressful condition. In a different *E. coli* strain, MG1655, Nolan Tronche (Ph.D. student, Laboratoire de Chimie Bactérienne, Marseille, France) investigated the roles of the transcriptional regulator YbcM produced by the defective prophage DLP12. Transcriptomic studies have shown that YbcM down-regulates multiple genes involved in bacterial motility while it upregulates genes involved in biofilm formation. These results suggest that prophage-encoded YbcM acts as a regulatory switch from motile to biofilm lifestyle in *E. coli*.

The impact of prophages on the ecology and evolution of their hosts was also more largely discussed in the keynote lecture of Anna Dragoš (researcher, Biotechnical Faculty, University of Lubljana, Slovenia). She illustrated the significant impact that Spβ-like phages have on *Bacillus subtilis* ecology with the example of spore formation, which is controlled by the excision of the Spβ prophage [26]. Dragoš and coworkers shed further light on the evolution and diversification of this family of phages by revealing that repeated cycles of sporulation can promote the recombination of Spβ with another prophage, phi3T, which is present in some *B. subtilis* isolates [27]. Prophages can therefore control the entry of bacteria in a dormant state, which is generally viewed as a prime strategy to survive stress, including phage infection. On the other hand, during his keynote speech, Alexander Harms (researcher, ETH Zürich, Switzerland) reported that the virulent phage Paride actively lyses antibiotic-tolerant *P. aeruginosa* cells that are in a dormant state. This work could have important implications in the fight against chronic infections that are highly refractory to antibiotic treatment [28].

Diving deeper into phage–host genome interactions, Amaury Bignaud (Ph.D. student, Institut Pasteur, Université Paris Cité, France) used the above-mentioned 3C method to compare physical genome interactions between *P. aeruginosa* and two of its phages. The first phage, PAK_P3, interferes with host transcription [29], while the other, PhiKZ, actively degrades bacterial DNA and produces a nucleus-like compartment for its replication [30]. The 3C approach revealed how these phages disrupt their host genome by its decondensation and further indicated that phage genome folding varies as their transcriptional program progresses. Also studying phage-induced modifications of the bacterial cell, Audrey Labarde (engineer, I2BC, Gif-sur-Yvette, France) used fluorescence and cryo-microscopy to observe the compartmentalization of DNA replication and virions assembly in *B. subtilis* cytoplasm during SPP1 phage infection [31]. She showed that SPP1 DNA is concentrated in a membrane-less compartment with empty procapsids distributed at the periphery while DNA-filled particles accumulate away from the DNA compartment. The talks of Amaury Bignaud and Audrey Labarde highlighted two different strategies employed by phages to spatially organise their replication and efficiently turn their hosts into viral factories.

Taking an even closer look at phages, Cécile Breyton (researcher, Institut de Biologie Structurale, Grenoble, France) presented how electron cryo-microscopy can help to understand how host recognition by phages triggers infection. She studied the structural organisation of the tail tip of *E. coli* phage T5 before and after the interaction of the bacterial receptor FhuA with the phage receptor binding protein (RBP) complex. Her work deciphered how the conformational changes occurring in the RBP after host recognition enable the opening of the phage tail tube, its anchoring to the membrane and the formation of a channel allowing the entry of phage DNA [32,33]. Beatriz Beamud (postdoctoral researcher, Institut Pasteur, Paris, France) also explored the topic of phage–host recognition. Her work tackled the challenging task of measuring the predictability of phage–bacteria recognition. Using >100 isolates of the capsulated bacterium *K. pneumoniae* and a collection of nearly 50 phages, she showed that most phages have a capsular specificity. The infectivity against a given isolate was accurately predicted by the capsular locus type of the host and the phage depolymerase enzyme. This work suggests the possibility of predicting phage–host combinations through the genomic analysis of phages and bacteria [34].

From phage-mediated regulation of bacterial processes such as spore or biofilm formation to molecular conformation of phage receptor binding proteins, this session dived into the close relationship between phages and their bacterial hosts.

### 2.4. Therapeutic and Biotechnological Applications of Phages

The final session of the meeting was dedicated to the development and optimisation of phage therapy. Key aspects included methodological advances in fundamental, preclinical, and veterinary settings as well as progress towards understanding the impact of immune cells and metabolites from the mammalian host on the biology of phage predation.

Prior to these scientific developments, a historical perspective on phage therapy was presented by the invited speaker, Claas Kirchhelle (researcher, historian of the University College Dublin, Ireland), who reconsidered the common definition of phage therapy as a “forgotten cure” confined to the former Soviet Union in the post–Second World War era. Against the argument that phage therapy was only recently re-evaluated on a larger geographical scale, he brought to light new documents dated from the 1940s to the 1980s. He showed proof of active production and use of phages for both bacterial typing and therapeutic purposes by public health laboratories, with a focus on the “Service des Bactériophages” (Institut Pasteur, Paris). The production, application and international dissemination of phage products were discussed, along with their recent decline after the finances and infrastructure of the service could not support the increasing demand. This research highlights the fact that studying neglected archives from public phage laboratories may help to face current challenges in phage therapy.

One of these challenges is the pharmaceutical production of therapeutic phages in compliance with the requirements of regulatory agencies. Frédéric Laurent (professor, Hospices Civils de Lyon, Université de Lyon, France) presented the development of the first French public platform for phage production and use in humans, under the PHAGEinLYON program and the PHAGE-ONE project [35]. The team performed an in silico screening of over 3000 clinical strains of *Staphylococcus aureus* to select fitting hosts lacking prophages and virulence factors. Next, phage amplification was experimentally optimised to produce high yields of three phages infecting *S. aureus*, resulting in pharmaceutical products suitable for clinical use after a complex but necessary purification process, quality controls and batch releases.

Another challenge in phage therapy is the need for products targeting a vast diversity of epidemic bacterial strains belonging to the same species. This can be achieved by employing generalist phages. Two speakers at the meeting presented their approaches to extend phage host range through evolutionary training, based on the Appelmans protocol [36]. Julien Lossouarn (research engineer, INRAE Micalis, Jouy-en-Josas, France) used a cocktail of four phages targeting the opportunistic pathogen *Enterococcus faecium*. Directed in vitro evolution over 15 passages broadened the infectivity of the cocktail from 3 to 7 (out of 8 tested) bacterial strains. Genomic analysis of generalist-evolved phages highlighted point mutations in genes encoding for their tail fibres, new positions of insertion sequence (IS), and recombinational events between different phages. The approach taken by Amandine Maurin (Ph.D. student, CNRS UMR 224 MIVEGEC, Montpellier, France) aimed at broadening the host range of phage PhiTB2C infecting *Salmonella enterica* serotype Tennessee. The ancestral phage could infect 3 out of 8 bacterial genotypes, and phage-insensitive bacteria rapidly grew. After seven passages, the phage expanded both its host range, by infecting all the tested bacterial genotypes, and its virulence, by suppressing the emergence of bacterial resistance. Molecular determinants of this adaptation included parallel mutations in genes encoding for the long tail fibres and for exo- and endonucleases.

While in vitro studies are crucial to optimise the safety and the therapeutic potential of phages, their efficacy also depends on the environmental conditions encountered within the organism to be treated [37,38,39]. Two speakers at this meeting presented data on the interactions among phages, bacteria and the mammalian host. Lionel Schiavolin (postdoctoral researcher, Université Libre de Bruxelles, Belgium) showed that phages infecting *Streptococcus pyogenes* behave differently in standard laboratory medium compared with an environment mimicking the human host. Sophia Zborowsky (postdoctoral researcher, Institut Pasteur, Université Paris Cité, France) investigated the determinants of immunophage synergy in a murine model of infection by *P. aeruginosa* [40]. By using neutropenic mice, she showed that phage PAK_P1 loses the curative effect displayed in wild-type animals, and that the level of neutrophils negatively correlated with the severity of bacterial infection. Unexpectedly, in macrophage-depleted mice, phage treatment was more effective in lowering the pathogen load compared with wild-type mice. These presentations highlight the need for awareness of host-related parameters that could alter the efficacy of phage therapy.

An experimental veterinary phage therapy approach was presented by Lorna Agape (Ph.D. student, INRAE, Université de Tours, France). Here, the prophylactic administration of a phage cocktail against *Salmonella enteriditis* led to reduced bacterial colonisation and fitness in a chicken model. Similarly, the pathogen load in the caeca was diminished by a therapeutic treatment administered 24 days post infection, showing promise for poultry farming and safety of the food chain.

### 2.5. Phage Research in Teaching and Public Engagement

This French Phage Network conference also highlighted science communication with a talk by researchers engaged in teaching phage research. Ombeline Rossier and Christophe Regeard (researchers, I2BC, Gif-sur-Yvette, France) presented a multi-unit teaching program for undergraduates of Université Paris Saclay to learn how to isolate and characterize environmental *Corynebacterium* phages. Phage hunting, beyond its own research interest, is a good pretext to practice environmental sampling, bacterial culture, DNA extraction, genome annotation and even electron microscopy. This very complete training led to a genome announcement note in preparation and allowed students to present their results in a young researchers’ symposium. These two speakers were not the only ones to involve students in phage isolation and characterisation. It was also via an undergraduate program that Alexander Harms coordinated the enrichment and the extensive characterisation of a collection of 80 phages that was made available to the research community [41].

Charlotte Brives (anthropologist, Centre Emile Durkheim, Bordeaux, France) studied the ecosystem—according to her own word—where phage research has evolved for decades. She was inspired by the work of Bruno Latour (1947–2022), and especially his book “The Pasteurization of France” (1983), explaining how Louis Pasteur (1822–1895) introduced the concept of microbes in French society. In her popular book “Face à l’antibiorésistance” (“Facing Antibiotic Resistance”), Charlotte Brives provided an overview of the societal questions raised by the growing need for phage therapy despite the lack of production units and regulatory framework. For instance, pharmaceutical companies are struggling to move from the “ready-to-wear” logic that was permitted by antibiotics and other drugs, while phage therapy would rather fall under the scope of personalized medicine. She also discussed the negative perception of viruses by the public, which could represent an additional hurdle limiting the development of phage therapy.

## 3. Conclusions

**Figure 2:**
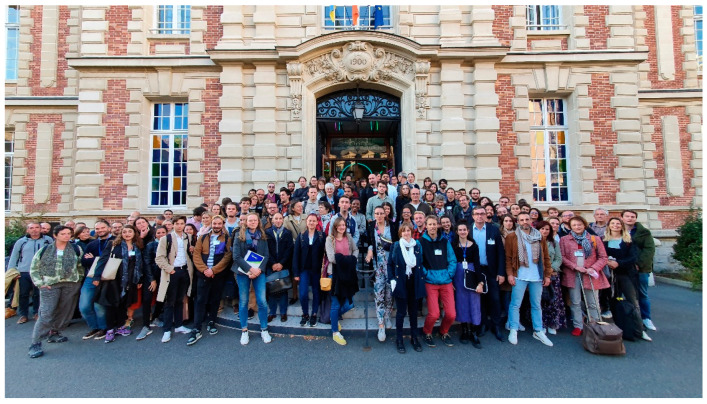
Attendees in front of the historical building at the Institut Pasteur.

With seven years of existence, the annual conference of the French Phage Network has become an unmissable event for French phage scientists but also for researchers from other countries across Europe and Africa. Keeping a moderate number of attendees (164, Figure 2) allowed informal discussions between the sessions and provided opportunities to establish new collaborations in a friendly atmosphere. Given the current excitement about novel defence systems and the still-growing demand for phage therapy interventions, there is no doubt that the phage research community will continue to be attractive to both students and senior scientists from other disciplines, ranging from mathematics and physics to human and social sciences, which overall will enrich the community. The next annual meeting of the French Phage Network will take place in November 2023 in Lyon.

## Data Availability

Not applicable.

## Supplementary Materials

The following supporting information can be downloaded at: https://www.mdpi.com/article/10.3390/v15020495/s1, Table S1: Posters presented during the seventh annual conference of the French Phage Network.
