# Supplementary material for: “French Phage Network” Annual Conference—Seventh Meeting Report
Source: Viruses. 2023 Feb 10;15(2):495. doi: 10.3390/v15020495 (PMC9966839; doi:10.3390/v15020495)
Supplement: Supplementary file 1 [file viruses-15-00495-s001.zip › viruses-2227453-supplementary.pdf]

"French Phage Network" Annual Conference - Seventh Meeting Report.

Olivier Schiettekatte[1], Elsa Beurrier[2], Luisa De Sordi[3] and Anne Chevallereau[4,*]

[1] Palais de la Découverte, Universcience, 75008 Paris, France
[2] MIVEGEC, Université Montpellier, CNRS, IRD, 34090 Montpellier, France
[3] Centre de Recherche St Antoine, Sorbonne Université, INSERM, 75012 Paris, France

[4] Institut Cochin, Université Paris Cité, CNRS, INSERM, 75014 Paris, France

*Correspondence: anne.chevallereau@inserm.fr

Supplementary information

Table S1. Posters presented during the seventh annual conference of the French Phage Network

Poster presenter	Poster title
Chloé Feltin<br>Unité de Pathologie Végétale, INRAE	Do phages of *Pseudomonas syringae* communities reflect the ecology and diversity of bacteria in apricot trees?
Karine Cahier<br>UMR 8227, IFREMER	Divergence in bacterial ecology is reflected by difference in population genetic structure, phage-predator load and host range
Sol Vendrell-Fernandez<br>Genetics of Biofilms Unit, Institut Pasteur	Repurposing a DGR-containing bacteriophage to study non-amenable *Bacteroides*
Yannick Labreuche<br>Station Biologique de Roscoff UMR 8227 LBI2M, IFREMER	Characterization of a T4-related vibriophage with broad host range
Hélène Batina<br>INRAE	*Erwinia* phages as biocontrol tools against fireblight disease in fruit trees
Aleksandra Otwinowska<br>Department of Pathogen Biology and Immunology, University of Wroclaw, Wroclaw	Insight into key structural points of capsule-targeting phage depolymerases specific to *Klebsiella pneumoniae* K63
Clémence Mouville<br>Institut Necker Enfants-Malades, INSERM, CNRS, Université Paris Cité	Molecular mechanisms of phage MDAΦ entry in *Neisseria meningitidis*
Mathieu De Jode<br>Sciensano	Current and future clinical phage product quality control in Belgium
Heloise Georjon<br>U1284 INSERM, Université de Paris	Evolution and conservation of antiviral mechanisms between Prokaryotes and Eukaryotes
Huaiyu Wang<br>Environmental Microbial Genomics Group, Laboratoire Ampère, Ecole Centrale de Lyon, University of Lyon	Determining the scale and rates of adaptation by methane-oxidising bacteria in response to in situ virus interactions

Ritam Das<br>Department of Life Science, Acharya Narendra Dev College, University of Delhi	A F4 cluster Mycobacteriophage Ritam007 with biofilm inhibitory activity and a unique lysis cassette
Nicolas Taveau<br>GreenPhage	Combining a Phage/Arbuscular Mycorrhizal Fungi-based protective formulation against the plant pathogen *Xanthomonas hortorum pv. Vitians*
Martine Boccara<br>Institut de biologie de l'ENS, UMR8197 CNRS	Progress in interferometric microscopy
Marianne Nicolas<br>Infectiologie et Santé Publique, Université de Tours, UMR1282, INRAE	Evaluation of a phage cocktail to prevent avian colibacillosis
Coline Meynard-Doumence<br>Centre de Recherche Saint-Antoine, Sorbonne Université, INSERM	Oxidative stress alters the interactions occurring in a community of intestinal bacteria and bacteriophages
Florian Tesson<br>IAME, INSERM, Université Sorbonne Paris nord, U1284, INSERM, Université de Paris	Systematic and quantitative view of the antiviral arsenal of prokaryotes
Maxime Decodts<br>Laboratoire de Matériaux Céramiques et de Mathématiques, CERAMATHS, Université Polytechnique Hauts de France	Bioceramics and Phage Therapy
Clara Douadi<br>Microbiote, intestin et inflammation, Centre de Recherche Saint-Antoine, Sorbonne Université, INSERM	Translocation of bacteriophages across the intestinal barrier: relationships with paracellular permeability and inflammation
Clara Torres-Barceló<br>Unité de Pathologie Végétale, INRAE	"Jack of all strains, master of all"? Phylogenetic host range of phages correlates with phage virulence
Clarisse Plantady<br>Pherecydes Pharma, Institut Cochin, CNRS UMR8104 INSERM U1016, Université Paris Cité	Identification of the receptors of four therapeutic phages used in human phage therapy
Théo Foutel-Rodier<br>Equipe Génomique des Vibrios, IFREMER	Fishing for phage lineages to understand their evolution
Marianne De Paepe<br>Micalis Institute, AgroParisTech, INRAE, Université Paris-Saclay	Mismatch repair and phage mutation rates
Luis Ramirez-Chamorro<br>Institute for Integrative Biology of the Cell, Université Paris-Saclay, CEA, CNRS	Host DNA destruction and the (subtle?) puzzle of phage T5 pre-early genes
Marie Titecat<br>U1286 INFINITE, INSERM, Université de Lille	Safety and efficacy of an AIEC-targeted bacteriophage cocktail in a mice colitis model
Ambre Moreau	Phage T5 encodes a new pathway for RpoS degradation, the master regulator of the general stress response in *E. coli*

Laboratoire de Chimie Bactérienne<br>Aix-Marseille Université, UMR7283<br>CNRS	
Caroline Lebrun<br>Laboratoire de Chimie Bactérienne<br>Aix-Marseille Université, UMR7283<br>CNRS	Isolation and characterisation of virulent phages against European *Xylella fastidiosa* subspecies
Julián Agustín Bulssico<br>Laboratoire de Chimie Bactérienne<br>Aix-Marseille Université, UMR7283<br>CNRS	Quantification of virus driven plaque expansion in real time
Marta Mansos Lourenco<br>Biodiversity and Epidemiology of Bacterial Pathogens Unit, Global Health Department, Institut Pasteur	Phages against non-capsulated Klebsiella pneumoniae: broader host range, slower resistance
Floriane Laumay<br>Institut des Agents Infectieux, Laboratoire de Bactériologie, Hospices Civils de Lyon	PHAGEinLYON -PHAG-ONE: Isolation and characterization of phages active against multidrug-resistant ST131 *E. coli* strains producing carbapenemases and/or extended-spectrum beta-lactamases
Mélanie Bonhomme, Mathieu Medina<br>Institut des Agents Infectieux, Laboratoire de Bactériologie<br>Hospices Civils de Lyon	Directed in vitro evolution of anti-*Staphylococcus aureus* therapeutic phages: host range expansion against multi-drug resistant Staphylococcus epidermidis isolates
Alexandra Sirot<br>3PHM UMR-S1139, INSERM, Université Paris Cité	Impact of faeces preparation methods for the study of the virome by NGS
Marion Coves<br>PROSE, INRAE, Université Paris-Saclay	Characterization of virus-host dynamics in anaerobic digesters under abiotic stress
Callypso Pellegri<br>Laboratoire d'ingénierie des systèmes macromoléculaires Aix Marseille Université, CNRS	Characterization of new molecular partners involved in filamentous phage infection
